# Diagnosing learning and teaching potentials – a cognitive linguistic analysis of conceptions of heart excitation

**DOI:** 10.3205/zma001437

**Published:** 2021-02-15

**Authors:** Mathias Trauschke

**Affiliations:** 1 Leibniz Universität Hannover, Institut für Didaktik der Naturwissenschaften (AG Biologiedidaktik), Hannover, Germany

**Keywords:** cardiac excitation, electrocardiogram, conceptual metaphor theory, model of educational reconstruction

## Abstract

**Purpose: **The analysis of difficulties in understanding the information content of an electrocardiogram revealed indications that these are also caused by misconceptions about cardiac excitation phenomena. Therefore, a re-analysis of these research data is intended to deepen the understanding that medical students have of cardiac excitation. Furthermore, the concept of excitation represented in academic textbooks will be examined from an educational perspective.

**Methods:** In order to diagnose learning potentials, a previous study on ECG comprehension collected statements from students using problem-centred, guideline-based interviews. These data were subjected to a re-analysis. The evaluation was based on a qualitative content analysis. Ideas of heart excitation and the underlying basal cognitions were analysed in the light of the Conceptual Metaphor theory using systematic metaphor analysis. Conceptual metaphors, which structure the understanding of this abstract fact, were identified. In a similar procedure, scientific ideas from textbooks were examined, too. The model of educational reconstruction served as the research framework.

**Results:** On the basis of the data from exemplary cases, it will be shown which subject-related inappropriate ideas students of human medicine can construct when dealing with the phenomenon of excitation in a cardiological context. For example, excitation can be misinterpreted synonymously with the extracellular potential differences responsible for the development of an ECG. Sometimes, excitation is even understood as the tone of the myocardium. Analyzing the educational potential of academic textbooks reveals possible barriers to understanding in that excitation is not clearly defined in terms of the de- and repolarization of heart muscle cells. Moreover, both students and textbooks show an inappropriate idea of repolarization.

**Conclusion: **The presented analysis of learning and teaching potentials offers the opportunity to identify difficulties in understanding with regard to an appropriate concept of cardiac excitation. It also helps to develop conclusions for educational interventions.

## Zusammenfassung

**Zielsetzung:** Bei der Analyse von Verständnisschwierigkeiten über den Aussagegehalt eines Elektrokardiogramms ergaben sich Hinweise, dass diese auch durch fehlleitende Vorstellungen über kardiale Erregungsphänomene bedingt werden. Durch eine Sekundäranalyse dieser Forschungsdaten soll daher das Verständnis von Medizinstudierenden über Herzerregung vertieft analysiert werden. Außerdem wird der in akademischen Lehrbüchern repräsentierte Erregungsbegriff aus didaktischer Perspektive untersucht.

**Methodik: **Zur Diagnostik von Lernpotenzialen wurden in einer vorausgegangenen Studie zum EKG-Verständnis Aussagen von Studierenden mithilfe problemzentrierter, Leitfaden-gestützter Interviews erfasst. Diese Daten wurden einer Sekundäranalyse unterzogen. Die Auswertung erfolgte durch qualitative Inhaltsanalyse. Vorstellungen über Herzerregung und die diesen zugrundeliegenden basalen Kognitionen wurden im Lichte der Conceptual Metaphor Theory mithilfe der systematischen Metapherntheorie analysiert. Dabei wurden konzeptbildende Metaphern identifiziert, welche das Verstehen dieses abstrakten Sachverhaltes strukturieren. In einem gleichartigen Vorgehen wurden wissenschaftliche Vorstellungen aus Lehrbüchern untersucht. Als Forschungsrahmen diente das Modell der didaktischen Rekonstruktion.

**Ergebnisse: **Anhand exemplarischer Fälle werden fachlich unpassende Vorstellungen aufgezeigt, die Studierende der Humanmedizin mit dem Phänomen der Erregung im kardiologischen Kontext konstruieren können. Erregung kann beispielsweise synonym zu den für das Zustandekommen eines EKG verantworten extrazellulären Potenzialdifferenzen fehlgedeutet werden. Mitunter wird unter Erregung sogar der Tonus des Myokards verstanden. Die Vermittlungspotenzialanalyse akademischer Lehrbücher zeigt insofern mögliche Verständnishürden auf, als dass Erregung nicht trennscharf hinsichtlich der De- und Repolarisierung von Herzmuskelzellen abgegrenzt wird. Überdies zeigt sich sowohl bei Studierenden als auch in Fachbüchern eine fachlich unpassende Vorstellung von Erregungsrückbildung.

**Schlussfolgerung: **Durch die vorgestellte Analyse von Lern und Vermittlungspotenzialen bietet sich die Möglichkeit, Verständnisschwierigkeiten hinsichtlich eines angemessenen Erregungsbegriffs zu identifizieren und Folgerungen für Vermittlungsinterventionen zu entwickeln.

## 1. Introduction

Interpreting electrocardiograms (ECG) is one of the central training contents for students of human medicine. A basic understanding of a physiological ECG is necessary for this. However, there are various professionally inappropriate, learning-inhibiting ideas in this respect that have been observed among medical students of higher semesters. For example, subjects are not able to describe the relationship between the course of the curve and the direction of excitation [1]. Identified difficulties in understanding are based, among other things, on inappropriate conceptions of cardiac excitation phenomena. Therefore, the findings discovered in the qualitative exploratory interview study on ideas of physiological ECGs [1] will be re-analysed in order to explicitly examine the concept of excitation in more detail. The research approach aims to identify misconstrued ideas about heart excitation. The reconstruction of ideas performed by using a neurocognitive linguistic theory of understanding [2], [3], [4] and systematic metaphor analysis [5] to analyse linguistic utterances from the test subjects. The essay presents four concept-forming metaphors on the basis of which students can misunderstand the processes of heart excitation. Furthermore, it is shown that similar metaphorical concepts are found in academic textbooks.

## 2. Theoretical approach – how to reconstruct and analyze conceptions

This work aims at the analysis of ideas of cardiac excitation processes. Here, the term “conception” is understood as an interpreted representation of mental experience, i.e. it is reconstructed from spoken or written utterances, which can be attributed to an individual in a context-specific way. Furthermore, conceptions are understood as a mental activity of a defined period of time which is significant and meaningful to the thinker, whereby both, the gradual process as well as the subjectivity [3] are highlighted.

However, according to the constructivist paradigm, conceptions are neither directly nor objectively accessible to researchers. It is only possible to draw conclusions about what is thought from spoken or written expressions. For the reconstruction of such subjective worlds of meaning a qualitative-explorative research approach is therefore chosen.

In order to reconstruct ideas about processes of heart excitation a suitable theory of understanding is required. As abstract facts as well as the biochemical processes of cardiac excitation are understood via “concept-forming metaphors” [6], the Conceptual Metaphor Theory [2], [3], [4] is used: According to the embodied cognition approach, we generate embodied ideas through interaction with our physical and social environment which form the core of the cognitions available to us. By unwillingly projecting such “cognitive primitives” [6], abstract facts can be mentally represented. Embodied ideas used for the cognitive exploration of abstract facts are called concept-forming metaphors. This cognitive-linguistic concept of metaphor must be clearly distinguished from the philosophical and everyday understanding of metaphor, since metaphors are traditionally described as consciously used expressions of figurative-poetic language [7]. Metaphors are rather physically manifested in the brain: Rohrer [8] was able to show, for example, that metaphorical understanding stimulates the same regions in the brain as the physical experience of the source area of the metaphor. When someone talks about understanding things that cannot be grasped, primary motor and somatosensory areas of the cerebral cortex, which are responsible for the movement of the hand and wrist, are activated in the brain of the listener.

**Figure 1:**
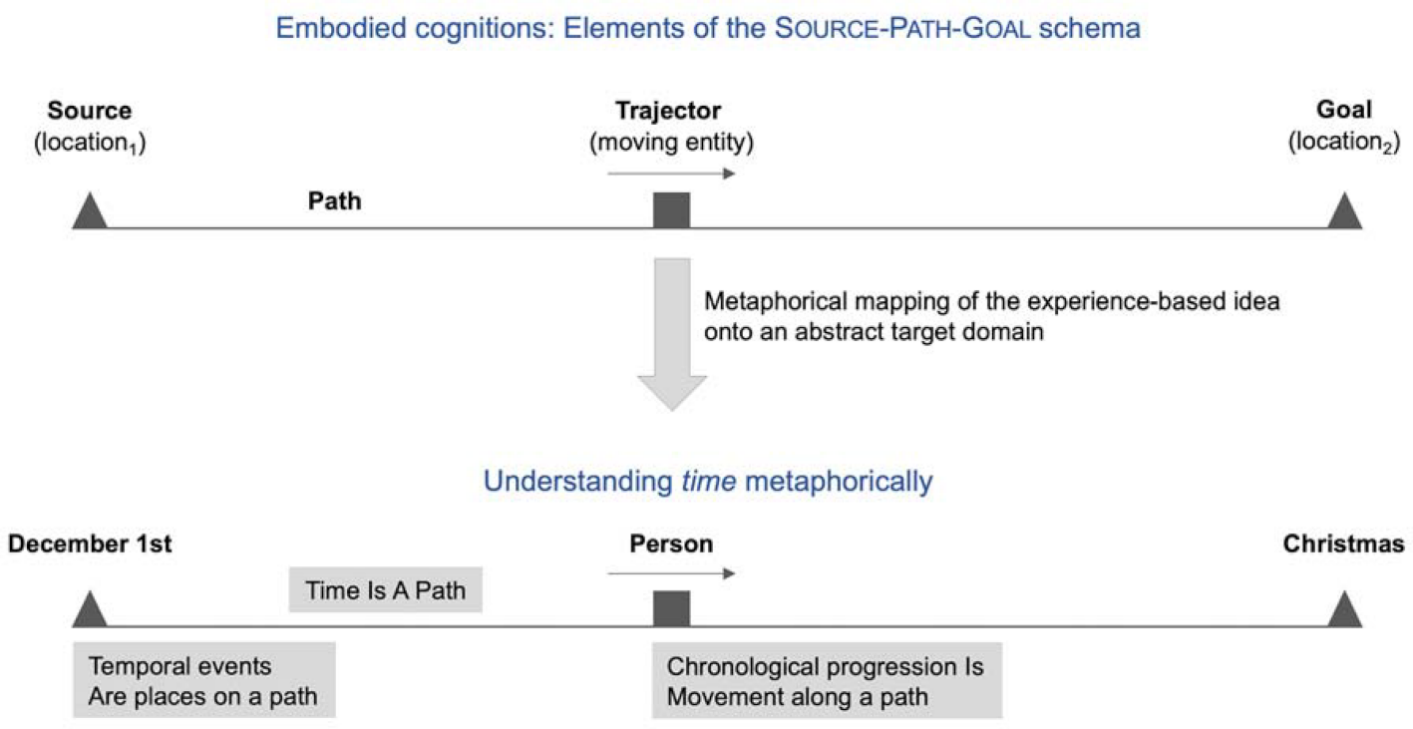
SOURCE-PATH-GOAL schema – embodied cognition and metaphorical projection in understanding temporal phenomena.

Among all the embodied ideas are a series of defined cognitive schemata [7], [9]. Taking the SOURCE-PATH-GOAL schema [9] as an example, everyday ideas are used below to illustrate how schemata can be used as concept-forming metaphors in understanding abstract facts (cf. also figure 1 (Fig. 1)). The following statement, for example, is directly understandable:

"Marie leaves the sandbox and walks across the lawn to the terrace."

According to Johnson [9], this is due to embodied ideas that we have been making since our earliest childhood by moving around in space. Physical movements or actions are usually linked to the covering of a distance. There is usually a starting point and a destination that we move towards as a moving entity. The SOURCE-PATH-GOAL schema is seen as an experience-based cognition that we have at our disposal and with the help of which the above sentence can acquire meaning.

Time, for example, belongs to a phenomenon that cannot be directly experienced. Temporary processes can therefore only be grasped imaginatively, for example through metaphorical projection of the SOURCE-PATH-GOAL schema:

"From now on, we must accept fewer appointments. The first Sunday of Advent is already past and Christmas is coming up. But we have not yet bought presents."

Hence, temporary phenomena can be represented mentally by using the embodied schema of directed movement [10]. Temporal events are metaphorically understood as places, time itself is understood as a path that has been (or still has to be) travelled. We understand ourselves as moving entities that move in a directed manner along a path. Time courses are metaphorically conceived as directed movements along a path [11].

In cognitive linguistics, numerous cognitive schemata have been described that enable the understanding of abstract facts [9]. Gropengießer [3] has shown a way to causally interpret the construction of ideas of abstract facts by working out concept-forming metaphors from linguistic utterances. Thus, an instrument for analysis is available for the diagnosis of incorrect conceptions. 

For better comprehensibility, table 1 (Tab. 1) explains the basal logic of the schemata that are later relevant in the analysis of the ideas of cardiac excitation phenomena and it illustrates their basic metaphorical projection using selected educational examples.

## 3. Research questions

Understanding an ECG requires, among other things, professionally appropriate ideas of excitation in a cardiological context. This research approach should contribute to the identification of possible misconceptions of heart excitation which can be constructed by students of human medicine or which can be identified in scientific representations. The following research questions are therefore of interest:

- Which ideas structure the concept of cardiac excitation of students of human medicine and which conceptual metaphors structure this understanding?
- What scientific ideas about cardiac excitation are constructed in academic textbooks?

## 4. Research design

### 4.1. Model of educational reconstruction 

The model of educational reconstruction (MER) [11] was developed as a theoretical and methodological framework for the planning, realization and evaluation of educational research. It aims at optimizing the teaching and learning of certain subject contents. This involves analysing learners' ideas of specific issues to identify difficulties in understanding (learning potential diagnosis). In addition, the teaching potential of scientific concepts listed in academic textbooks is examined (teaching potential analysis). In a reciprocal comparison, the respective ideas regarding commonalities and differences are examined. Based on the findings it is feasible to create possible options for the design of learning environments and teaching interventions (educational structuring). 

#### 4.2. Methods 

A re-analysis of research data is carried out to diagnose learning potentials. According to Bortz and Döring [12], such a selective secondary analysis of specific research data has to be conceptually distinguished from a meta-analysis of comprehensive research findings. The findings are taken from a previous study of medical students' ideas of the ECG [1]. In the course of this analysis, medical students (n=10) were interviewed individually using problem-centred, guideline-based interviews [13]. In addition, a developed intervention was tested with the help of individually conducted teaching experiments [14] using thinking aloud protocols [15]. The students were primarily asked about the electrocardiogram, but were also encouraged to express their opinions on the phenomenon of excitation (e.g: What do students imagine excitation to be, how does it originate, how do test persons imagine excitation in the heart in the course of time, which aspects of excitation are shown in the ECG and how are they shown). All test persons were informed in detail about the methodological procedure and have given their consent. The data were anonymised, a matching of the quotations to corresponding persons is not possible due to pseudonyms.

In the context of this second access, the focus lies on the reconstruction of the concept of excitation that students constructed in the single interviews conducted. The available interview data (here: statements and videographed gestures) are again examined by using qualitative content analysis [16]. According to Mayring's “scientific method” [17], the results are interpreted against the background of the research question and the identification of categories. The approach of inductive categorization [17] is used here, in which ideas are interpreted using systematic metaphor analysis [5]. For this purpose, all utterances are extracted that refer to the process of heart excitation – either in principle or specifically in the context of the ECG. The identified conceptual metaphors of excitation are denoted in by X Is Y. The capitalised Is means is understood as. X stands for an abstract fact that is understood by an experience-based conception Y. The identified cognitive schemata, which underlie metaphorical thinking, are presented in small caps. The following table 2 (Tab. 2) shows how statements, conceptual metaphors and underlying cognitive schemata are separated.

In this way, all identifiable metaphors for the concept of excitation are collected, interpreted and assigned to the respective, anonymised test persons.

In order to ensure the transparency of the interpretation and the associated intersubjectivity [18], all explications are linked to concrete anchor quotations.

The teaching potential analysis is carried out in a similar way. Qualitative content analysis [16] is used by first summarizing meaningful text passages or illustrations from academic textbooks (ordered statements). This is followed by the reconstruction and interpretation of scientific ideas (explication), also using systematic metaphor analysis [5]. Academic textbooks are suitable for analysis because they present scientific ideas (here: heart excitation) especially addressed to students [11]. A total of three common textbooks are used for this examination. The focus was on literature which can be usually found in textbook collections of medical libraries.

## 5. Results

The following results are structured according to the investigation steps of the MER.

### 5.1. Learning potential diagnosis 

For the sake of clarity, exemplary quotations from selected test subjects are listed (ordered statements) and analysed from a teaching perspective (explication). In the course of this, the metaphor analysis is carried out, from which metaphorical understanding (individual structuring) is derived. The underlined statements are of particular relevance for explication. They represent key words or text passages on the basis of which metaphorical concepts of excitation are derived. In this way the comprehensibility of the interpretation is ensured.

#### 5.1.1. Excitation concept A: excitation can rise and fall (VERTICAL-ORIENTATION schema)

Johannes_4 (103-129): “When I measure something in millivolts, that is a voltage (...) Well, I would say that the excitation is highest here (pointing to the maximum of the R-wave), or rather the voltage.”

Johannes_4 (147-158): “This is the P-wave. The other is also the P-wave – the ascending and descending part of the P-wave. This is then the descending [part of the P-wave]. Since this is the atrial excitation, [I interpret this as] the rising and falling excitation of the atria.”

The constructed idea can be traced back to the metaphorical use of basic cognitions about verticality. In the light of the conceptual metaphor theory it becomes clear that the abstract fact of excitation is understood to be a state that is variable on an imagined vertical scale. In this respect, the anchor quotations indicate that excitement can “rise” and “fall” or reach a “highest point” in John's imagination. The conception of rising and falling excitation is based on the metaphorical projection of the VERTICAL-ORIENTATION schema – a fact which is not directly comprehensible is then understood by an "orientation metaphor" [2]. 

From a teaching perspective, this idea proves to be problematic, since for John excitation and voltage have the same semantic meaning. For the test subject, the maximum of the R wave is both the point of “highest voltage” and the moment of “maximum excitation”. The test person overlooks the fact that an extracellular potential difference is measured or mapped in the ECG, but not the excitation of the heart tissue itself. Excitation is thus erroneously equated with the potential difference externally derived in the ECG (which can indeed be imagined as falling and rising). Trauschke [1] was able to show that misconceptions of the ECG arise in this way. Similar difficulties in understanding excitation are also shown by Kathrin's statement:

Kathrin_13 (300): “In any case, with the sinking of the P-wave no loss of voltage occurs, but simply the excitation of another part of the atrium. The excitation does not become less, but still increases and the voltage still decreases, I don't understand that yet”.

The test subject obviously does not succeed in separating the processes of excitation from the extracellular potential differences shown in the ECG, either.

##### 5.1.2. Excitation concept B: excitation is a directionally moving entity (OBJECT schema; SOURCE-PATH-GOAL schema)

Johannes_4 (397-402): “[I imagine] that the excitation follows the same path that it has taken through the heart muscles, i.e. once from A to B and again from B back to A – spatially speaking.”

Johannes_4 (508-516): “If you take it very strictly in a spatial way, this part of the S-wave must still be there because when the excitation has reached the apex of the heart here, the excitation moves relatively quickly to the ends [shows with both hands a path from the lower end of the heart to the upper ends of the two ventricles].”

The non-physical is often understood physically by using substance metaphors [2]. The anchor quotations suggest that excitation is also understood as a kind of moving, quasi-material entity that moves through the heart. The basal cognition used is the OBJECT schema. Johannes imagines excitation passing through heart muscle cells from a defined point A and returning to a destination (point B). According to our everyday experience, the traveling of a path can only be attributed to a material entity. The conception is supported by gestures, in that the imaginary course of excitation (cf. Anker's quotation 5) is shown by a parabolic upward movement of the hands from a starting point (=tip of the heart). The abstract phenomenon of excitation is thus also made comprehensible by the SOURCE-PATH-GOAL schema. 

Jan also imagines excitation as a quasi-material entity (excitation is an object) that is moving through the heart tissue. The abstract process of excitation transmission is also mentally structured by the SOURCE-PATH-GOAL schema because excitation is understood as a trajectory passing through start and end points.

Jan_7 (189-206): “My idea of excitation that spreads out across the atrium [is that] I have the image of the anatomy of the atrium in front of me. I see a room in front of me and then I imagine that the excitation is there and then it starts somewhere and I visualise the excitation in such a way that I change the colour of this area. Then I [imagine] that this is simply a spreading, [that] the excitation, this colour, [spreads] until the whole atrium has this colour.”

From the perspective of education, another idea that hinders learning is presented here. Its focus is not on the functionality of excitation, but only on the local change of an entity. The central scientific idea of locally spreading depolarisations and the electromechanical coupling that ultimately results from them are not considered, however. Furthermore, it is not appropriate to imagine excitation going back and forth because it is not possible to distinguish between de- and repolarisation in this way.

##### 5.1.3. Excitation concept C: excitation is muscle tone and enables blood movement (ENABLEMENT schema)

Johannes_4 (429-452): “Excitation is what we actually say, when a muscle is excited we always mean that it contracts. When I think about at what point in time the excitation or the maximum excitation occurs, I would say this is the case when the heart muscle is at maximum contraction and that would be in the ejection phase, in systole, when it tries with all its strength to eject the blood (...) So intuitively I would say that maybe [on the R-peak] the whole thing is maximal.”

Excitation is thought to be the heart's ability to pump. The variable manifestation of excitation is equated to the muscle tone (“contractility”) of the heart. Therefore, the contractility of the heart is thought to be the ability to let something possible transfer into something existing. This idea is based on the use of the ENABLEMENT schema [9]. Seen from an educational perspective, this is a misleading idea. This concept of excitation is unaware of electrochemical processes on cell membranes. The test subject does not imagine an appropriate relationship between excitation and contraction.

##### 5.1.4. Excitation concept D: regression of excitation (DECREASE schema)

From an educational point of view, the idea of regressing excitation is relevant, which will be shown below. Annalena introduces the idea that excitation increases and decreases (diminishes). This conception is considered to be experience-based. Cognitions of becoming smaller or larger, which, according to Riemeier, stem from the everyday understanding of growth, are used metaphorically [19].

Annalena_8 (56-65): “I would say [that] here (on the ST route) the excitation is greatest, because the ventricles are then completely excited and then is the transition. So there the excitation decreases again with the regression of excitation.”

Annalena_8 (555-574): “[If I wrote down the main statement of the ECG,] I would now think about what I would like to have in there. (...) So the ECG measures the electrical excitation propagation and regression in the atria and ventricles of the heart. Now I'm thinking, that it shows that in millivolts.”

From an educational point of view, the idea of regressing excitation – after all used as an antonym for excitation propagation in academic textbooks [20], [21], [22] – is misleading. The term propagation refers to the spatial distribution of entities. When an entity spreads out, it occupies more and more space or area. The conception of excitation propagation is therefore scientifically appropriate, since a process (depolarisation) is locally propagated along heart muscle cell membranes.

However, the concept of regression is inappropriate, it is contrary to an adequate understanding of spreading repolarisation: The term regression refers to the decrease in phenomenological manifestations. For example, symptoms of a disease can recede or subside. A swelling or an organ (e.g. uterus after delivery) can also recede. However, this notion is not suitable for an appropriate description of the renewed reversal of the membrane potential through ion fluxes (repolarisation) and its local spreading. Spreading (something spreads) and regression (something becomes smaller, decreases) are non-suitable linguistic terms to describe the twofold reversal and respective conduction of membrane potential changes by ion flows (de- and repolarisation). 

An impeding influence on learning can also be seen in Kathrin's statements. She speaks of excitation regression and even of “re-excitation”. Thus, it becomes clear that the basal cognition of regression is not suitable for describing the electrical processes in the heart in a relevant way.

Kathrin_13 (225-249): “Well, it [the excitation] already diminishes, but that does not mean that the excitation is already complete and is going to *regress*.”

Kathrin_13 (286-294): “The ECG shows the excitation of the heart, i.e. atrium and myocardium, and is based on the anatomical relations. The re-excitation is then also seen in the last wave. There is a physiological reason for all this. So I wrote: the ECG shows the excitation of the heart, i.e. the atrium and the myocardium, based on anatomical relations and in the last part you can see the re-excitation of the myocardium.”

An impeding effect on learning can also be seen in Kathrin's statements. She speaks of excitation regression and even of “re-excitation”. Thus, it becomes clear that the basal cognition of regression is not suitable for describing the electrical processes in the heart in a relevant way.

In summary, it is shown which ideas could be identified within the groups of test subjects (see table 3 (Tab. 3)).

##### 5.1.5. Limitations

The reconstructed conceptions have no claim to completeness. Likewise, they cannot be representative which is due to the dependence on the qualitative approach necessary for the interpretation of subjective worlds of meaning. Thus, a generalization beyond the sample is not possible. However, the main value of the research results is the gaining of insights into potential misconceptions and to describe them in a clear-cut manner. 

A further limitation is based on the methodological approach. It cannot be ruled out that further statements could be triggered in problem-centred and guideline-based interviews, if the interview guideline focused mainly on the concept of excitation (in the interviews carried out this was only a partial aspect, because it was primarily a matter of recording ideas about the physiological ECG). Moreover, it is possible that students would identify these misconceptions if they were explicitly confronted with them in a retrospective survey. Therefore, the findings have to be declared as context- and situation-dependent.

#### 5.2. Teaching potential analysis 

The research task consists of the methodically controlled, systematic investigation of scientific statements from an educational perspective [11]. These scientific ideas are subjected to an educational (not a scientific!) analysis. In this process, their potential for teaching is examined with regard to misleading ideas, i.e. whether and to what extent the scientific representations can promote or impede teaching or learning. For the sake of clarity, the teaching potential diagnosis only looks at examples. 

##### 5.2.1. Excitation concept B: excitation is a directionally moving entity (OBJECT schema; SOURCE-PATH-GOAL schema)

Johannes and Jan construe inappropriate ideas of the transmission of excitation. They imagine excitation as a quasi-material entity, that moves back and forth in the heart, without describing its functionality. A linguistic and figural representation (see [20], image 14.18 to cardiac excitation, where excitation is understood as a quasi-material entity) used by Silverthorn [20] must indeed be viewed critically with regard to such a substance-like notion of excitation (spreading violet colouring). Since the facts of the changing electrical properties are not dealt with, this type of external representation lacks a central subject-related component: The process of excitation is represented insufficiently. Due to the two-dimensional colouring, however, excitation can be perceived by readers as a quasi-material fluid which is distributed within the heart.

Furthermore, aspects of repolarisation are not represented visually. In contrast, this is done in an analogue visualisation in Silbernagl and Despopoulos [21]. Under the heading “Excitation propagation in the heart” (195, fig. C), repolarisation is also included. However, this makes it clear that two fundamentally divergent concepts of excitation propagation are presented:

1. excitation propagation = propagation of depolarisation
2. excitation propagation = propagation of de- and repolarisation

From a teaching point of view, this subject-related inconsistency is questionable because learners are confronted with fundamentally different ideas.

##### 5.2.2. Excitation concept D: Regression of excitation (DECREASE schema)

Considering scientific statements from an educational perspective reveals that even in scientific sources misleading everyday conceptions can be identified [23]. This can be illustrated by the use of the term “regression”, which is not appropriate to the subject. Fleischmann and colleagues [22] explain in chapter “8.1.1 Heart excitation” the “spreading of excitation” (394). In this regard, they distinguish between excitation propagation and degeneration.

(394): “Precise knowledge of the propagation and regression of excitation in the heart is a prerequisite for understanding the ECG”. (...) “The excitation reaches the AV node very early, before the atria are fully excited. (...).” “The excitation then spreads very quickly via the excitation conduction system of the ventricles. ”

(400): “The P-wave indicates the propagation of the excitation through the atria, (...)”. 

 (395): “The excitation regresses from the apex of the heart (...)”.

 (401): “The T-wave signals the regression of the excitation in the ventricles, (...)”.

The propagation of excitation, i.e. the continuing depolarisation between neighbouring heart muscle cells, is basically a scientifically appropriate term. However, the term can promote a quasi-material idea of excitation (5.1.4). The concept of regression, on the other hand, must be viewed critically from a professional and educational perspective. As already explained, the term regression is used to describe the basal, experience-based cognitions of becoming smaller (DECREASE schema). Nevertheless, the metaphorical use of this idea of regression is inappropriate to describe a process reciprocal to excitation propagation (strictly speaking, containment would be the semantically appropriate counter term to propagation). But exactly this would be needed from a physiological point of view because the process depicted as regression is conceptually identical on a molecular level (renewed charge reversal by ion currents). Depolarisation (reversal of a membrane potential) and repolarisation (renewed reversal of the membrane potential to the initial state) would have to spread equally according to this functional logic - an idea apparently preferred by Silbernagl and Despopoulos (see [21], figure to excitation propagation). However, this is exactly what is imprecisely represented by the word pair excitement propagation versus excitation regression. The regression of excitation is thus basically a misleading term. 

##### 5.2.3. Limitations

Since this diagnosis of potential for teaching has an exemplary character, no claim is made to completeness. Nevertheless, it should be stated that the analysis of more textbooks could lead to more empirical findings and thus, the sample of gathered conceptions could be enlarged. Due to the qualitative research approach it can also be stated that the data are not representative. 

Historical sources are sometimes examined in the context of the teaching potential diagnosis as this provides insights into the history of concepts [11]. No such in-depth form of examination was carried out. It could, however, prove useful in order to clarify the term “excitation”, which is used vaguely in the cardiological context, in a more decisive way with regard to its origin and meaning.

## 6. Discussion and educational approaches

**Figure 2:**
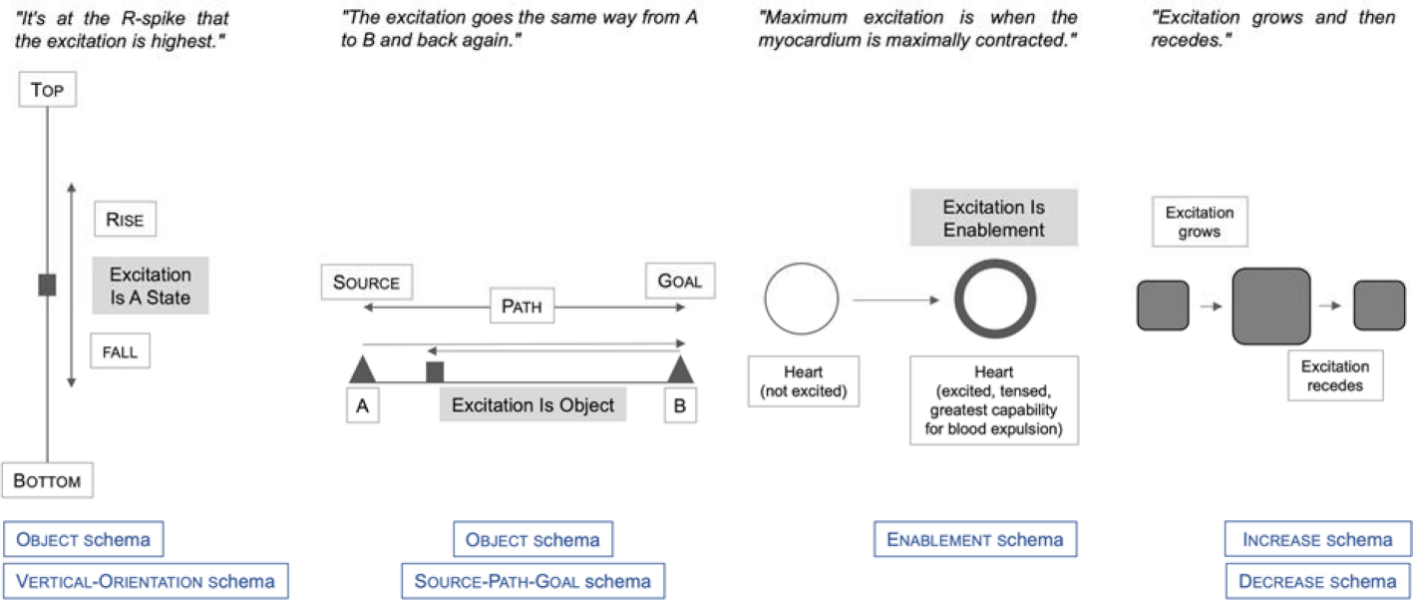
Cognitive schemas used metaphorically in understanding cardiac excitation.

The diagnosis of learning potential shows how misguided ideas can be identified using a neurocognitive-linguistic theory of understanding. Regarding this, four conceptual metaphors structure the ideas of heart excitation of the test subjects (see also figure 2 (Fig. 2)).

1. VERTICAL-ORIENTATION schema: Excitation is understood as a rising and falling state (and equated with extracellular potential differences), which is shown in the ECG curve.
2. SOURCE-PATH-GOAL schema, OBJECT schema: Excitation is understood as a quasi-material entity that moves back and forth in a directed manner within the heart. The continuing depolarizations that occur in this process are not taken into account, nor is electromechanical coupling considered as being the subsequent process.
3. ENABLEMENT schema: Excitation is understood as the tone or the tension of the myocardium.
4. DECREASE schema: Excitation is understood as an electrical phenomenon that can regress (decay, decrease). In one case, an additional idea of re-excitation could be identified.

As the exemplary teaching potential analysis presented here shows, the idea of excitation as a quasi-material entity moving within the heart can also be found in academic textbooks (SOURCE-PATH-GOAL schema, OBJECT schema). It also applies to the conception of excitation regression (DECREASE schema). It therefore becomes clear that scientific ideas are sometimes based on the same experience-based cognitions as learners’ ideas. However, this research approach does not allow any conclusions as to whether and to what extent these conceptions contribute to the formation of the inappropriate subject-related perceptions of students or if they are even causally related to them. However, it is reasonable to assume that these textbook concepts do not contribute to the construction of adequate concepts. Nor can they cause a re-learning towards adequate ideas. The step in the study in which we analyse the teaching potential of textbooks, thus fundamentally clarifies the benefit of a subject-related educational analysis of scientific ideas because aspects that hinder learning can be identified herewith. Furthermore, the analysis of teaching potentials provides – here as an example – the insight that professional concepts are not clearly communicated across textbooks. For example, it is unclear to learners whether the processes of de- and repolarisation can be carried out under excitation, or whether excitation can only be described as depolarisation of the heart muscle cells. Such professional uncertainty, represented by corresponding diagrams (see [20], image to excitation in the heart, where excitation is understood as a quasi-material entity and [21], figure to excitation propagation), is deplorable from a teaching perspective as it poses an obstacle to learning.

In the following, the misleading ideas that were recorded are discussed. Approaches for the planning of teaching approaches are also outlined.

### 6.1. Excitation versus differences in extracellular potentials 

Knowledge of the individual conceptions provides further indications of difficulties that students may have in understanding the physiological ECG. In this context, the misconceived idea that an ECG represents the increase or decrease of excitation (VERTICAL-ORIENTATION schema) is of importance. On the one hand, the actual cause for the formation of the ECG curve (extracellular potential differences) is disregarded. On the other hand, this way no idea can be constructed that the ECG curve represents the direction of the excitation transmission. 

In order to facilitate learning, a suitable approach to avoid or overcome this inappropriate conception should highlight the relationship between electrical excitation, difference in extracellular potential and curve shapes in the ECG. A sophisticated illustration can be found in Pape, Kurtz and Silbernagl [[24], 205, Fig. 5.31]. Here excitation is illustrated as a process of charge redistribution along the cell membranes of heart muscle cells. At the same time, it is clearly shown that this molecular process is not directly depicted in the ECG. 

Furthermore, the processual nature of excitation is indicated. Although the ion ratios are not shown, the resulting charge ratios are represented. This is also to be regarded as conducive to learning considering the difficulties in understanding (6.2 and 6.3) discussed below.

#### 6.2. Excitation is a process and not a moving substance

In the research of science education it is well-known that abstract phenomena such as energy or heat are understood as quasi-material phenomena employing substance metaphors [25]. In this respect, the conception of excitation as a substance-like entity, as captured here, corresponds to the expectations of metaphor theory. Even in scientific textbooks, our thinking and the associated use of language hardly allows us to avoid substance metaphors. Nevertheless, an attempt should be made, especially in textbooks, to emphasise the processual nature of the excitation process (transferred de- and repolarisation). This is precisely what students should learn in order to grasp the connection between excitation and the differences in extracellular potentials caused by it and thus be able to understand the fundamental principles of ECG development. 

With regard to visualisations in academic textbooks, illustrations which depict excitation propagation without reference to the changing charge conditions (see [20], image to cardiac excitation, where excitation is understood as a quasi-material entity) and thus convey both a substantial and an incomplete concept of excitation propagation (cf. above all the ideas of the test subject Jan) should therefore be avoided. 

Apart from the molecular aspects not taken into account, the idea of a substance-like excitation, which is spreading, also falls short with regard to causal relationships since the connection between electrical excitation and mechanical coupling is not explained. Linguistically or figuratively expressed notions in which the arrival of excitation is the goal are therefore not suitable concepts in the sense of scientific causality. In graphic illustrations, the completely depolarised heart should therefore not be shown as the target, but rather as the starting point for initiating muscular contractions. In this way, the conceptual void in the test persons' ideas could be prevented.

#### 6.3. Misunderstanding excitation as a mechanical state

As it turns out, the degree of contraction of the myocardium can be falsely conceptualized by the term excitation. This may be categorised as a pronounced weakness in understanding in advanced students. However, in the light of the theory of embodied cognitions this conception can be explained easily. Muscle tone can be experienced in the real world. The term excitation is also used every day to describe the feeling of excitement or tension. It is therefore understandable that in individual cases, in which professional discussions of the subject probably took place on a rather superficial level, the physical experience of tension structures the understanding of heart activity. Aiming towards learnability, however, a pronounced obstacle to learning becomes apparent in two respects: First, excitation is not understood as the redistribution of ions and the resulting differences in membrane potential. Second, excitation is not understood as a process at all, but as a state. A basic approach to teaching should therefore a) focus on excitation being a process (cf. also 6.1) and also b) focus less on the organ level (see [20], image to cardiac excitation, where excitation is understood as a quasi-material entity) and more on the cell and molecule level. It is precisely at this system level of living things that the processes of ion redistribution (excitation) and the resulting mechanical processes (contractions of the sarcomeres) can be represented in a much more useful way. 

#### 6.4. Excitation cannot recede

From an educational point of view the usage of the term “regression of excitation” for a reciprocal process for the propagation of excitation has to be discussed. The term excitation propagation refers to excitation as a change in membrane potential (depolarisation) and the local transmission of this process across adjacent cells. The word regression, on the other hand, neglects the aspect that repolarization is also a process of spreading. The term is not suitable to show that the original membrane potential is restored after excitation. However, excitation – i.e. the depolarisation of a membrane beyond a threshold potential – cannot regress (= decay, decrease). What is meant and what is more appropriate from a professional point of view, is rather the concept of reversal or restoration, which could also be represented metaphorically: the TWIN-PAN-BALANCE schema [9] is a suitable basal cognition for this. Accordingly, we have basal cognitions of balancing, for example derived from playing on a seesaw. The seesaw has two load arms and a rotation axis and we have usually had physical experience of how these load arms behave in relation to weight and movement. The ideas of balance gained from this interaction are used to understand abstract facts metaphorically, e. g:

- Lara is much more emotionally balanced today than yesterday.
- The level of punishment and the extent of crime should balance each other out.

The schema is subjectively appropriate in that two variable states of the membrane potential – depolarised and repolarised – are set alternately. The entire system can virtually assume two states, each of which can be transferred back to the other. Electrical excitation phenomena can also be communicated via this scheme. Corresponding statements can basically be constructed as follows:

- Excitation: Ion currents along the cell membrane cause a reversal of the charge distribution (depolarisation). The cell can be called excited.
- Excited cells act as a stimulus on surrounding muscle cells and also imitate depolarisation there. In this way, the process of excitation is spatially transmitted within the heart tissue.
- By spreading the redistribution of ions, the charge conditions are reversed again. The achievement of the original membrane potential associated with this is called repolarisation.

## 7. Conclusions

Teachers often do not consider the effectiveness of teaching impulses on the cognitive learning process of learners. Pfäffli [26], for example, criticises that university teachers derive their teaching process from subjective ideas about learning. Thus, teaching often takes place as a pure sequence of monologic lectures contrary to recommendations of constructivist theories of teaching and learning interactions. Knowledge about possible difficulties in understanding therefore fundamentally broadens the perspective of teachers because more focus can be placed on the results of planned learning situations. 

With regard to a professionally appropriate understanding of heart stimulation, the metaphorical ideas of students show obstacles for learning. As the analysis of the teaching potential analysis shows, similar non-suitable conceptions can also be found in academic textbooks. This suggests that subject-specific ideas should be critically and constructively examined from a teaching perspective in order to revise any misleading representations of scientific concepts that may hinder learning.

In principle, this re-analysis provides further insights into how inappropriate ideas about cardiac excitation can lead to the difficulties in understanding the ECG curve [1] previously identified. This includes the conception of excitation as a substance-like entity or as a state of muscular contraction. The idea of excitation regression identified in students' statements and academic textbooks was found to be fundamentally inappropriate. It therefore seems advisable, in preclinical teaching, to precisely describe cardiac excitation phenomena at the molecular level as a process of de- and repolarisation by ion flows. Especially in iconic representations of the transmission of de- and repolarisation, substance metaphors should be avoided because they neglect the core scientific aspects on the electrochemical level.

The misguided ideas shown can also be used to contrast them in courses with subject-related correct concepts and thus create cognitive conflicts as a starting point for learning. In addition, students can be presented with inappropriate ideas in seminars for critical review and correction, thus enabling a deeper examination of the subject matter.

As is made clear in this paper, the educational view of subject matter also leads to findings that are relevant for teaching and learning. This includes the analysis of the misleading description of excitation regression in textbooks, which does not provide learners with an understandable representation of the molecular processes on heart muscle cells. The model of educational reconstruction, in conjunction with conceptual metaphor theory, has therefore proved to be a fruitful research design for analysing ideas about subject matter. In the future, ideas that university lecturers have of heart excitation and the significance of the ECG would be of research interest. In addition, it could also be examined which individual teaching experience they have with students.

## Acknowledgements

Special thanks go to Ute Heine for scholarly exchange. I give also thanks to the reviewers for their constructive suggestions on the manuscript.

## Competing interests

The author declares that he has no competing interests.

## Figures and Tables

**Table 1:**
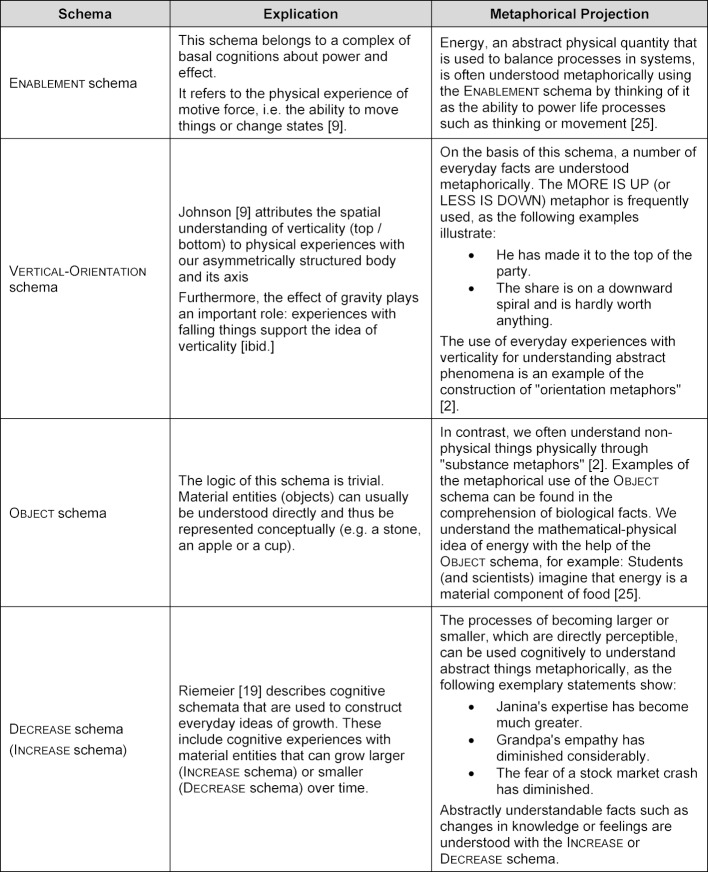
Cognitive schemata and their metaphorical use in understanding abstract facts

**Table 2:**
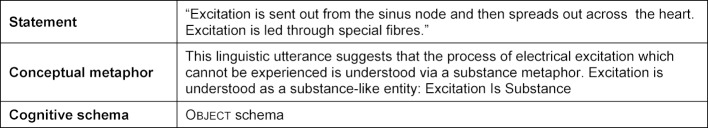
Derivation of conceptual metaphors

**Table 3:**
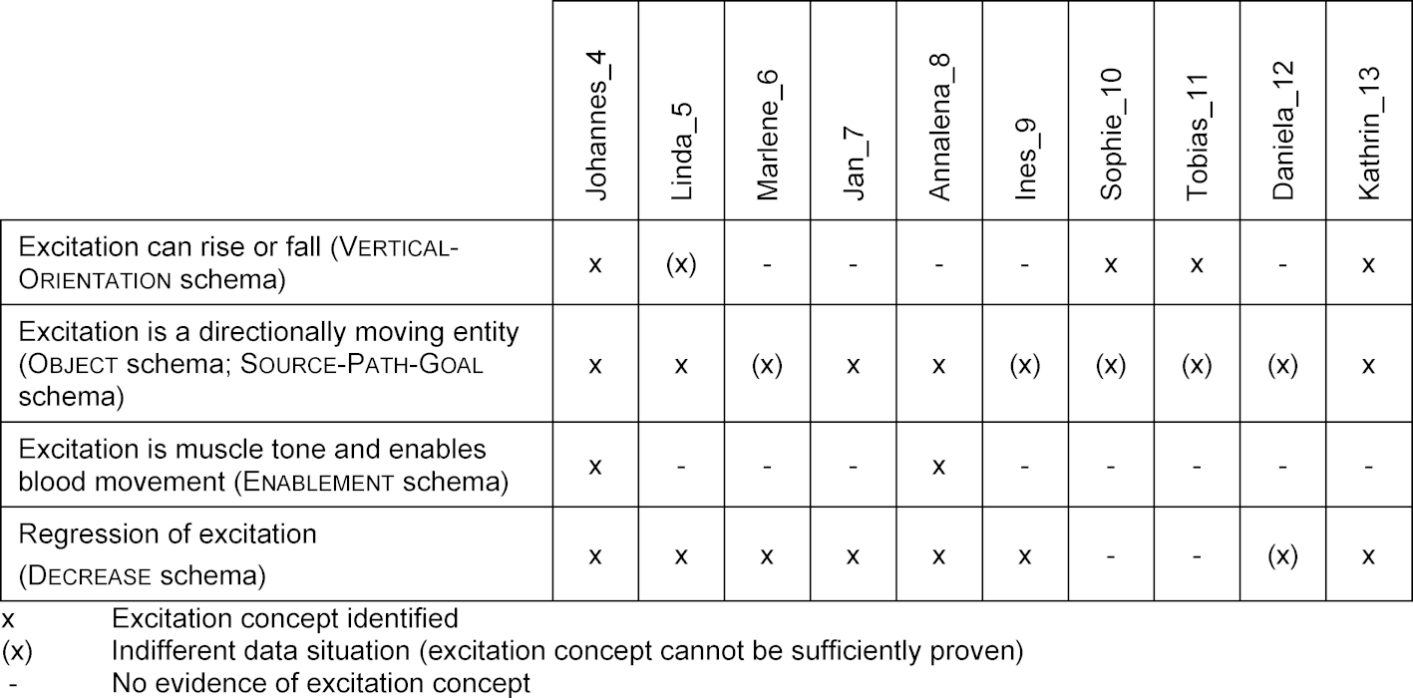
Concepts of excitation identified within the sample
